# The architecture of the hybrid lab: Spacing graphene research

**DOI:** 10.1177/03063127221114721

**Published:** 2022-08-06

**Authors:** Albena Yaneva

**Affiliations:** University of Manchester, Manchester, UK

**Keywords:** laboratory design, science architecture, nanoscience research, graphene, scientific practice

## Abstract

Building on and renewing a long line of STS research of lab materialities, this article sheds light on a type of architecture organized around the ‘heroic agency’ of a new nanomaterial, graphene. It examines how design responds to the dynamic and multi-applicational ambitions of contemporary science. An ethnographic study of the National Graphene Institute in Manchester allows us to see how the building’s design has epistemic and social effects, how different spatial arrangements facilitate the shaping of research habits and mediate various rhythms of lab work. Key features of this hybrid lab are: first, a shifting balance between public and private places, with a prevalence of collective activities; second, its capacity to reinforce epistemically and socially the conditions of visibility, by emphasizing the work of making research practice visible; third, its distinctive way to speed up research, often by slowing down the circulation of people and things. All these features make the hybrid lab a unique spatial articulation of a new cultural order of innovation.

Walking around the campus of the University of Manchester in the early 2010s, I watched a large five-storey building taking shape. Cranes and diggers, scaffolding and dust, construction companies and workers with different coloured high-vis vests crowded that part of campus. Slowly, the distinctive blocky silhouette of the building, designed by the London-based firm Jestico+Whiles, appeared. It stood out in a dazzling black that, with its unique shape and sizable volume, broke the monotonous pattern of red brick buildings. Approaching the building, I could begin to see the sign – ingredients of a formula language (E, =, m, νF2) – engraved on the veil, with the subtle reference to the hexagonal-lattice of carbon atoms. Completed in 2015 and now full of life, six years after construction, the dust has settled and the workers have moved onto another site down the road.

This building hosts the National Graphene Institute (NGI) (Figure 1) and was raised following the ambition of the university to capitalize on the work done by two Nobel Prize winners in Physics – Andre Geim and Kostya Novoselov – on the isolation of graphene. Graphene is the foil of nanotubes, whose properties have been known for decades, but its extraction as a single layer with distinctive electrical properties and strength occurred in 2004. This marked the start of the process of isolating this first human-made 2D material. The applications of graphene have yet to be explored, and the new building serves as an incubator to this end by bringing together scientists and commercial partners under one roof (currently more than 250 people). The swift pace of graphene research in Manchester follows a tree-like development: The NGI functions as the trunk of this intertwined branching, extending to the £60m Graphene Engineering Innovation Centre (GEIC) designed by Rafael Viñoly (GEIC, completed in 2018), and the £105m Henry Royce Institute for advances materials research and innovation completed in 2020. A ‘graphene city’ has emerged and continues to reshape Manchester’s urban ecology.

**Figure 1.**
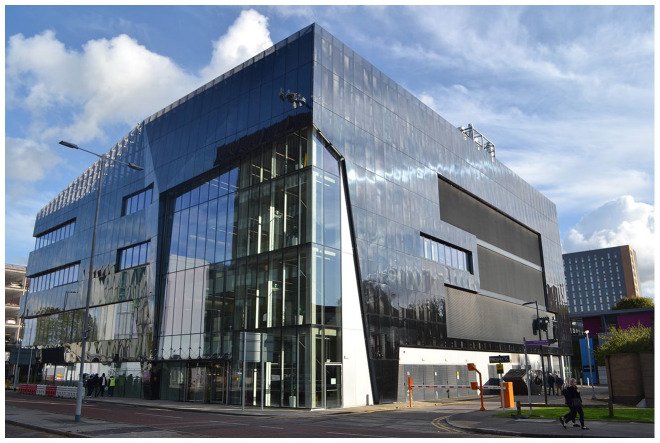
The National Graphene Institute, Manchester, UK.

The NGI building draws the attention of thousands of visitors as a showcase of British cutting-edge science (Novoselov & Yaneva, 2020). Its exceptional features include a clean room, designed with a specific focus on graphene and other 2D materials; this room is spread across two floors connected by a clean lift. There are also a variety of open space laboratories and specialized labs with grey spaces for storing equipment, and a number of social spaces and atria, roof terrace and garden, to ensure quality ‘breakouts’ and promote communication. The NGI contributes to UK’s role at the forefront of the commercialization of graphene.

As I walk along, I wonder: What kind of practices lurk behind this mysterious dark veil? How do scientists inhabit this ‘temple’ of twenty first century science? How does its design matter to graphene research? To answer these questions, I will engage in a close analysis of the material architecture of this building while simultaneously providing insights into its hectic laboratory life. First, though, I examine how issues of lab design have been tackled since the inception of laboratory studies.

## How design matters for laboratory life

The pioneers of lab studies engaged in meticulous explorations of science in the making to understand the cognitive and social dimensions of scientific experimentation, the fabrication of facts and the visualization of invisible structures. Though they often drew attention to the materialities of laboratories, for the most part the architecture of the scientific buildings in which their studies unfolded generally remained neutral backstages. None of the specific design features of Louis Khan’s acclaimed Salk Institute complex appear to matter in the ethnographic account of science in the making of Latour and Woolgar (1979). For Lynch (1985) the laboratory is a ‘locatable and definite set of rooms and facilities’, important as a setting only to the degree to which it is sketching the ‘ethnographic context’. He does not draw attention to specific spatial arrangements as relevant to the cognitive activities of neuroscientists. Another pioneering author of lab studies, Knorr-Cetina (1981), draws a generic picture of the modern lab as ‘a local accumulation of instruments and devices within a working space’ (p. 4), which gains specificity depending on the types of objects, apparatuses, furniture and samples accumulated. There is a striking contrast, in this wave of sociological studies of labs, between the context-specific contingences of the scientific activities they recount and the highly generic and abstract portraits of the lab which remains a neutral canvas of frantic activities, a tacit stage for ethnographic actions.

Tracing the practices of high energy physicists, Traweek (1988) provides the first account that draws attention to the spaces of science: buildings, libraries, canteens, wide or narrow shapes, windowed or windowless offices, cluttered or spacious rooms. Her extensive ‘inventories’ and meticulous mundane descriptions of the labs contain intriguing remarks on the materiality and aesthetics of these spaces (grey metal or glass, furnishing and surfaces and façades) and how the spatial practices of physicists reveal their scientific identities and the hierarchy among them. Although her ethnography, similarly to Lynch’s, begins with a ‘touring of the site’, the spaces she tours have more architectural features, textures and colours that the neutral lab plant described by Lynch, the two-wing blueprint depicted by Latour and Woolgar or the stack of drawers and glassware sketched by Knorr-Cetina. There is an incipient interest in the spatial and temporal patterns of scientific work for they illuminate the lab’s social and epistemic reality.

In the 1980s and 1990s, some of the most explicit discussions of the physical and social settings of experimentation and the siting of knowledge-making practices as contributing towards the practical solutions of epistemological problems came from historians of the early modern period (e.g. Shapin, 1988). Taking a ‘localist turn’ (Shapin, 1998), science studies gradually shifted attention towards the local production of meaning related to the urban context of labs, the way experimentation expands its given spatial nexus (Forgan, 1989; Shapin & Shaffer, 1985) and the spatiality of techno-scientific practice (Gooday, 1998). Tackling the interrelation of architecture and science, Hannaway (1986) compares the plans of two chemistry labs: the Danish astronomer Tycho Brahe’s castle lab Uraniborg, especially designed by architects to serve as an observatory and accommodate a chemistry lab, and the chemical lab of Andreas Libavius, both parts of dwellings. He argues that in both cases the architectural design expresses the scientific ideas and a certain understanding of the role of scientists and their civic responsibilities. Scrutinizing the lab plans and elevations, Hannaway goes into specific details (types of rooms, colours, furniture or instruments and their specific arrangement, storage rooms, atria) to argue that the plans reflect the ideological roots of the new modes of scientific life. Each lab placement has symbolic meaning in addition to functional purpose. Taking an even closer look at these plans, as architectural, not as representations of certain ideology, Shackelford (1993) reminds us that architectural plans are not just rhetorical devices, but reflect the logic imposed on the use of space by practical considerations: access to light, fuel, ventilation, heavy equipment, sheltering, maintenance and technical work.

The growing interest among historians of science in both the symbolic meaning and the practical aspects of the material spaces for science gradually began to carry over into studies of present-day labs. It resulted in several studies that addressed the design of physics and chemistry laboratories (Galison, 1997a; Hofmann, 2011; James, 1989; Rentetzi, 2005), the siting of biology labs as negotiating different boundaries with nature (Kohler, 2002), the scientific design of modern operating rooms as embodiment of control (Schlich, 2007) and the collaborations between visionary scientists and visionary architects (Leslie, 2008).

The ‘localist turn’ also led to a more explicit engagement of sociological science studies with the field of architecture and urban studies and a more sustained interest in the design of contemporary science buildings. This happened in the 1990s, which saw a wave of applications of molecular biology and the arrival of huge volumes of genomic and transcriptomic data from automated sequencing and DNA microarray technologies, as well as the publicly funded Human Genome Project with first tests of gene therapy on human patients. This moment of consolidation and expansion of molecular biology and biotechnology resulted in new lab buildings. Since the end of the 1990s several scholars focused on architecture’s role in the shaping of scientific cultures and identities (Galison & Thompson, 1999; Gieryn, 1998, 1999). Gieryn, in particular, carried out the most continued and rigorous empirical work on the relationship between contemporary science and architecture. Basing his findings predominantly on biotechnology labs, he argues that ‘the social structure of biotechnology is shaped by choices made during the design’ (Gieryn, 2002, p. 36); the complex nexus of knowledge and space has a significant impact on the credibility of scientific claims (Gieryn, 2006, 2008). Others tackled the local and spatial circumstances of science making as crucial ingredient in establishing the credibility and the status of knowledge (D. Livingston, 2003) and explored the impact of urban fabric and infrastructure on the production of scientific knowledge (Dierig et al., 2003).

The 2000s saw STS scholars expanding their methods, commonly used to study science, to the field of architecture (Houdart & Minato, 2009; Latour & Yaneva, 2008; Yaneva, 2005, 2009), following a programmatic article by Callon (1996). At the same time, architectural theorists also began referring to the epistemological framework of science studies (Frampton & Moore, 2001). Yet, in spite of this cross-fertilizing dialogue and exchange of methods between the two fields, when architectural scholars deal with scientific buildings, they often ignore the socio-material complexity of science in the making. Similarly, whenever STS scholars tackle the situatedness of science, they often do not engage extensively with the specific design features of lab architecture. As a result, there have been very few studies of laboratory buildings that house newer scientific practices since 2000, in spite of Gieryn’s powerful plea for more studies on how lab design contributes to the modulation of scientific research.

## Localized science, global world

Building on this long line of STS research on the materialities of science, I follow a ‘fifth wave’ of studies on the place-specific nature of science, that corresponds to a moment when globalized science is at the same time highly emplaced: ‘research happens at identifiable geographic locations amid special architectural and material circumstances’ (Henke & Gieryn, 2008, p. 353). It joins a handful of recent studies of lab spatiality that have paid particular attention to the space-making practices of scientists.

Mody’s (2005) laboratory ethnography of material science pays close attention to sounds in experimental spaces, which create situated knowledge. Reinforcing the importance of laboratory ethnography in scientific workspaces, Stephens and Lewis (2017) argue that attending to the rhythms of day-to-day work of scientists and the flows of matter better allows capturing their work as accomplished through interaction, set in and through spaces and rhythms. Similarly, tracing the complex relationship between spatialities and ‘science in the making’, Aroles and McLean (2019) trace how space matters in the everyday work of evolutionary biologists and argue that the different rhythmicity reshapes the direction of the scientific inquiry and the articulation of knowledge. Shifting attention to the design and planning processes behind scientific buildings, Vinck and Zarama’s (2011) ethnography of a nanoscience lab follows how spatial arrangements are shaped before the building’s construction and continue to be heterogeneous, fragmented and fragile after its completion; in the course of laboratory life researchers and spaces are mutually transformed.

However, the present study is different from previous ones in a number of ways. First, in focusing on a building entirely dedicated to the explorations of a newly isolated material, this study situates lab users’ spatial experiences within a cutting-edge area of contemporary science. Graphene research is now one of the hottest areas of study for physicists. ‘Each scientific period has its own “heroes”’, explains NGI’s lead scientist Kostya Novoselov, and, after carbon nanotubes, fullerenes, GaAs, high-temperature superconductors and many others, ‘graphene is the “hero” now’. Graphene is a simple material, easy to obtain and at the same time offering rich technological promises:

Indeed, in graphene we have a unique combination of properties, which are not seen together anywhere else: conductivity and transparency, mechanical strength and elasticity. Graphene can successfully replace many materials in a great number of existing applications, but I would also like to see things going in the other direction, with the unique combination of properties found in graphene inspiring completely new applications. (Novoselov, 2010, p. 121)

Second, due to the application-driven character of graphene science, research at NGI requires a specific social and cultural organization, involving a range of shared spaces for material scientists, engineers, chemists, physicists and people from industry. The NGI is a ‘hybrid’ lab, where hybridity refers not only to the melding of different disciplines and specialties (a feature studied extensively by STS scholars in the 1990s), but also to the entanglements of human and nonhuman participants in the spatial choreographies of lab life. The building is bestowed with the important role of reflecting and accomplishing a distinctive approach to research organized around the ‘heroic agency’ of graphene; its architecture affords different rhythms of applied research.

Third, there is something about graphene research that an ethnography of the NGI building is in a particularly strong position to capture. Such an ethnography, in following the rhythms of inhabitation, and in tracing the intimate connection between the pace of research and its material spatialization, provides a situated understanding of economic and other pressures that drive graphene research to move faster and faster. It is the building’s design that facilitates, by speeding up and slowing down, the development of graphene research.

Fourth, recent studies of lab architecture have often highlighted non-research spaces, from cafes to temporary dens to informal meeting rooms to walkways (Klonk, 2016), whose area ratio has grown considerably since the 1990s. These have been deemed important for socializing among researchers and as places where increasingly distended networks of players (top-quality investigators and industrial partners) can gather and plot. While the science lifestyles shaped as a result of active engagement with such features of lab architecture have received attention (Kaji-O’Grady et al., 2018), this study of graphene research presents a case for returning to the framings of late 1990s and early 2000s lab studies that showed the constitutive and performative role of architecture and building design in and for how science is practised. Exchanges of ideas and recipes often start at the lab bench in the clean room, in open labs or in the grey room. Thus, in what follows we will scrutinize various spaces that matter for the course of research (from labs, to gas rooms, to atria) and continue to hone our understanding of how design becomes an explicit and pervasive factor in the quest for scientific knowledge.

To what extent is architecture significant for globalized science? How, when and under what circumstances do specific spatial arrangements contribute to the shaping of new research habits and thought paths, and enable various rhythms of lab work? How do distinct spatial choreographies facilitate different epistemic and social practices related to graphene research? How is the work of spacing performed through the material architecture of the lab? Before engaging with these questions, let us unpack the specificity of the hybrid lab.

## Hybrid labs and their ethnographic exploration

According to Galison, as physics is no longer organized around the ‘modernist’ axes of steam and electricity or the ‘late modernist’ technics of ‘electronics’, the lab from the 1970s to the late 1990s is ‘postmodern’ in its ‘increasing reliance on highly sophisticated data processing at every stage: from design, to simulation, to experimental runs, to the processing of data’ (Galison, 1997b, p. 1129). This postmodern lab excels at creating massive collaborations, with shared sets of practices between disciplines and ‘trading languages’ aimed at solving problems in the borderland. If we were to extend Galison’s typology of labs, what would define the specificity of the new generation of scientific buildings (Joyce, 2004; Moe et al., 2013; Kaji-O’Grady & Smith, 2019) from the early twenty-first century onwards? Thrift (2006, p. 292) argues that these buildings are ‘performative’ machines ‘meant to manipulate time and space in order to produce intensified social interaction so that all manner of crossovers of ideas can be achieved’. Their distinctive features include: some form of public display of science, design intended to stimulate interdisciplinarity, porosity for scientists and information to constantly flow, transparency, all of this encouraging a ‘buzz’ of continuous conversation oriented to ‘transactional knowledge’ that contributes to innovation (Thrift, 2006). Such buildings have affinities to airports and shopping malls, all shaped by logics of global capital (Gieryn, 2008). While Thrift’s account captures well an architectural trend in the development of these new ‘temples of interdisciplinary science’ and outlines their specific role as innovation incubators, changing the business of invention in the fabric of capitalism, it fails to acknowledge the reality of the science that unfolds within their premises. In fact, Thrift’s list is neither derived from nor supported by his own ethnographic research of these labs. Building on Thrift’s analysis and engaging in a micro-sociological study of the importance of space in biomedical work, Stephens et al. (2008) characterize scientific buildings as having a ‘performative architecture’ capable of reflecting and framing social actions by aligning the rhythmicity and work patterns of multiple disciplines.

The NGI offers an extreme instance of ‘performative architecture’ and helps outline the profile of a new contemporary type of lab related to the dynamic and multi-applicational nature of science, the *hybrid lab*, with some distinctive features. It maintains a shifting balance between public and private places with the prevalence of collective activities in both experimental work and informal communication. It reinforces epistemically and socially the conditions of visibility by rendering scientists observable, and placing an additional emphasis on the work needed to make research visible. In addition, speed runs through the veins of the entire building: Huge elevators transport giant machines, there is a constant flow of supply of gases, large corridors facilitate the swift movement of people and equipment to labs, and those labs can be easily retrofitted. The material architecture speeds up the circulation of people and things and generates more hybrid entanglements.

To understand how design matters for multi-applicational science, I studied the NGI building in Manchester between 2017 and 2019, which was two to four years after it was built and during a period when it gradually entered into a steady-state phase of functioning. I base my findings on in-depth interviews with scientists, facility managers, clean room and lab technicians, administrators, porters and house service staff (thirty interviews), thus interrogating the collaborative practices of science production without forgetting the less prominent voices. Additionally, the account builds on a two-year long ANT-inspired ethnographic study in different parts of the building, to allow capturing on-the-ground spatial experiences and a range of material agencies. The aim is not to hear scientists judge the functionally of the building, as if it were a machine (working or not) as this often happens in post-occupancy studies (Gieryn, 2002), but to rather witness the rhythm of lab dwelling, the concrete spatial attachments, the speeds of work and various spatial choreographies of lab life.

Isolated and protected environments set barriers for an anthropologist to access and freely move around in the spaces. To circumvent these restrictions and gain a better understanding of how the building works, I took ethnographic walks with the interviewees, asking them to recreate their daily trajectories and the specific ways of engaging with the different features of the building (sixteen walks). This allowed me to develop insights into the different spatial routines that form the core of the laboratory life at the NGI. The spontaneously organized ‘show and tell’ sessions and the lab ‘tours’ that allow access to the setting are ethnographically convenient (Lynch, 1985). Ultimately, ‘a different sense of buildings comes from seeing them as “walk-through” machines’ (Gieryn, 2002, p. 41). Through the walking tours I was able to visit the clean rooms, wander the viewing corridors, sit in shielded labs and witness the buzz of busy Friday afternoons, when more than a hundred researchers and industry people storm the top floor to attend the famous graphene seminars. They allowed me to question people’s attachments to the building and take numerous photographs to explore the different material arrangements, equipment settings and inscription techniques that matter for their work.

In what follows, I trace one exemplar daily trajectory through the NGI building, as the young scientist Lewis Le Fevre goes from the open-plan lab (stop 1) to his specialized lab (stop 2), through to the grey room (stop 3), then down to the social spaces with writeable black walls (stop 4) and back to the specialized lab. One of many possible trajectories, it exemplifies a variety of spatial arrangements that form parts of graphene research on a daily basis. These stops can be viewed and visited in any order, as they are not linearly arranged sequential steps aligned in a straight movement. They allow me to take a closer look at the places (their materiality, arrangements and specific equipment) and to capture the rhythm of experimentation, exchange, connectivity, maintenance and servicing of these facilities. Taking these stops will shed light on a range of spatial experiences (both positive and negative) and epistemic effects that cannot be captured on a floor plan or a section of the building. And if a floor plan of a science building presents ‘a physicalized architecture of knowledge’ (Galison, 1997a, p. 785), this trajectory exemplifies the physicalized architecture of applied research.

Moreover, there is no single time or single space to be witnessed in Lewis’s trajectory. As Latour (1997, p. 178) argues, ‘we should not speak of time, space, and actant but rather of temporalization, spatialization, actantialization (the words are horrible) or more elegantly, of timing, spacing, acting’. Scientists at work rely on the subversion, disjunction, displacement, rescaling, translations, associations and crossing-over of relations between spatial, actorial and temporal features. In the multiplicity of interactions with equipment, samples, nanoparticles, heavy machines, walls, floors, transparent partitions, tests and benches, all having their own timing, spacing, goals and ends, new relations are formed. Space is actively dissected and observed, folded and unfolded. It emerges as a socio-material construct in the *work of spacing*. As they engage with the complex spatial design of the building, scientists are not more *in* space than they are in time. Thus, our analysis also unpacks the space-times of graphene activities, speeding up and slowing down, to pace again.

### Stop 1: The open-plan lab

Let us follow Lewis, a postdoctoral researcher working on energy storage devices. Crossing the first floor, he enters the open lab. The open-plan design contributes to the accessible and open nature of all spaces. As senior scientist Rahul explains, ‘the transparency afforded by the extensive use of glass forces communication among users and makes it easier to share knowledge’. Indeed, glass prevails and connects the clean room to the open lab, the offices to the atria. It allows people to observe huge pieces of equipment and gowned scientists busy with sample preparations. This open-plan space allows us to witness the power of design to facilitate the shaping of ‘adjacencies’ and resolving proximities (Shoshkes, 1989).

Lewis greets Andrey, a senior scientist, who is working in his office with a large door window overlooking the open lab. Andrey’s students are working in the open lab doing electrical characterization by measuring very low currents and very high resistances or thermal characterization by measuring with a laser flash to determine the thermal conductivity of materials. While they are working, they can easily see their supervisor in his office, and also witness other peers being active in the clean room; they also notice Lewis going through the open lab and greeting Andrey. Glass allows the simultaneous sound separation of working zones and the visual connection between different working dynamics. On the other end, the open lab has also a glass connection to the atrium where social spaces with writeable walls are located. This visual connection facilitates the flow of people and things. Light travels from the outside through the outer windows. Passing through the offices and the large glass interior windows, the light glides into the open lab and also reaches the clean room. Other large windows can bring light from the atrium around the open lab. This reinforces the idea of visibility as a condition of research efficiency: instant communication, easy access to equipment, recipes and processes and quick assistance and adjustment of equipment when needed.

This porous and transparent architecture speeds up the course of research. No time is wasted: From his office, Andrey keeps an eye on the work in the open lab and intervenes when needed. His students can witness the sample preparation process in the clean room and chat with Lewis while he is passing by. None of them is alone or secluded; the collective effort is ubiquitous. Yet the open architecture can also have a disadvantage: The glass windows and doors allow researchers and NGI administrative staff to be seen at all times when working in their offices. Senior scientist Sarah complains that it gets very noisy, and she sometimes cannot concentrate as they are constantly interacting: ‘[I]t’s hard to tell people, “I need some space. Don’t come in my office!” when they can see that you’re in there.’ Behind the see-through glass doors and windows, research is visible, its steps observable. The various pulses of graphene work can be witnessed at all times. And that is what Lewis can see while walking and talking to colleagues on the first floor. The open plan material architecture performs a spacing that intensifies the rhythm of graphene research by enhancing the quality of connections between scientists, students, preparation and characterization processes, different technologies and results.

### Stop 2: The energy lab

The building is ‘concentrated on laboratory space’, as Rahul defines it. There are eighteen specialized labs (chemistry, metrology, prototyping, microscopy, composites, furnaces, membranes, Raman spectroscopy, industrial labs, etc.). Compared to the large clean rooms with multiple users (approximately seventy, from different research groups and industry teams), which are wholly managed by the technical support team, the standard laboratories have fewer users (typically one or two principal investigators, accompanied by assistants, postdocs and grad students) and are ‘centred around one area of excellence’. Labs change easily from one type to another and users swiftly change their working sites. This double flexibility allows scientists at the NGI to easily change teams and perform any spatial modifications of their labs. Space follows the pace of research. Walls and partitions are ready to engage in a dance of endless re-configurations.

Hidden behind a heavy door on the second floor is the Energy Lab. This is where Lewis typically spends his days. He has a background in physics and is currently working on solar cells. Focusing on the chemistry and design of large energy storage devices like batteries and super-capacitors, his work could be placed between blue-sky fundamental research, on one side of the continuum, and the industry level, on the other.

**Figure 2.**
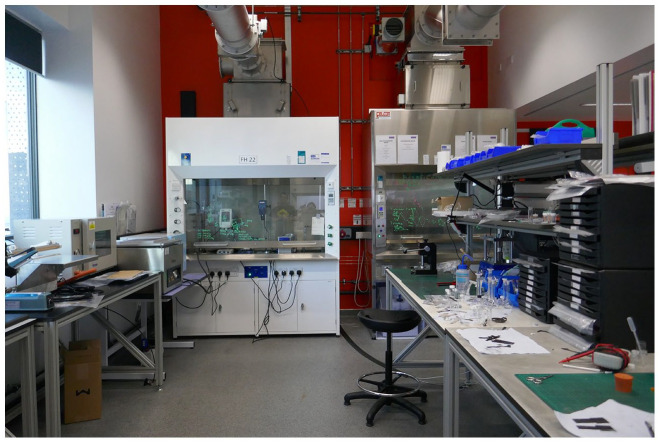
The energy lab.

The equipment is new and has the highest specs available in this area of research (see Figure 2). Everything looks immaculate. The intensive rhythm of work is seen only in black spots on the white cupboards; graphene makes its presence known through this grey powder. There are seven registered users for whom the energy lab is their primary lab, and they do all of their research in there. Other researchers visit the lab occasionally to use some of the equipment; in total, the energy lab had twelve lab users in 2017 and around twenty users in 2019.

The lab is designed to have stacks of equipment on top of each other, which makes for a more efficient use of space. A lot of new equipment was purchased in the past couple of years, as the funding increased between 2017 and 2019, and companies, especially British ones, have become much more interested in the work of the NGI energy team. The lab swims in light; in fact, an entire wall of windows connects the city with the researchers working here. Lewis explains, ‘in the morning you have much more light, compared to chemistry labs where you have one or two small size windows, but never a wall of windows’. The light improves the researchers’ well-being without interfering with their experiments as ‘none of the experiments are light sensitive’. Parts of the wall are painted in a joyful orange colour reflecting the daylight.

In contrast, the chemistry lab where Lewis had previously worked, in the Chemistry Building on campus, was very different. There he shared a dark space with twenty-five other researchers. ‘I don’t know how to describe it. It felt more cramped with big things. It felt like you were constantly walking over things or people to do anything. The space there was all being used up by other people and myself, and equipment, as well.’ Comparisons with this working space makes Lewis appreciate the new energy lab here at the NGI. He spends at least seven hours a day here, and only two hours in his office in the Chemistry Building (built in 1967) – most NGI scientists have labs or offices in other buildings on campus. He comes straight to the lab in the morning and sets some experiments. He can leave a running experiment, or start a new experiment for some analysis and then leave it running for an hour, two hours or even a day. Then he goes out of the lab to the grey room, the gas room or to the roof terrace. Yet, ‘[i]f I only have twenty minutes to wait, I’ll start doing analysis on one of the five communal computers we have in the lab.’ All of the equipment is run from a computer, but Lewis or any other researcher from that lab can simultaneously use the computers for data analysis, as long as no one else needs it to start another experiment. Speed is something that can be witnessed in this daily lab rhythm as we follow Lewis. In the Chemistry Building, everything is slow: ‘If it’s Monday, you thought of an idea, you wanted to do an experiment, sometimes you’d have to wait until Thursday before you can use the equipment.’ No time is wasted in waiting at the NGI; experiments promptly follow insights. The larger space accommodates more pieces of up-to-date equipment that accelerates the tempo of experimentation. The entire organization of the lab space outlines the importance of speed for graphene research.

Another way to speed up the course of research is at the level of chemical analysis. There is a system for analysis that can run multiple samples at once. In the Chemistry Building, Lewis can only run one sample at a time and wait for weeks while it completes, whereas here he can run several samples at the same time. As they have become much busier, they now have two machines for multi-scale testing, which allows him to test thirty samples at a time. Thus, numerous samples are analysed simultaneously and the chances to get good results multiply as well. The proximity of computers and experimental set-ups intensifies the discussions. When Lewis and his group have an experiment running, they all gather around the screen and discuss it together, trying to establish whether ‘they see something strange’ or ‘if the experiment is not doing what we thought it would do’. Most of his colleagues in the lab work on the same project and discussions on results and experiments unfold at any moment. As they deal with application-driven research, speed matters. As that particular rate at which scientists and sampling, apparatuses and testing come together, speed is ingrained in the specific material lab arrangement just as it is ingrained in the structure of the building.

The energy lab is full of written scripts spread on surfaces and walls meant to facilitate communication. These scripts bear the traces of discussions, shared arguments and fun: bets on experiments, different to-do lists, instructions, etc. On the glass surface of the fume hood are formulae and ‘instructions of how to do the testing’. As Lewis says, ‘it is actually easier to show people how to do things by visualizing it rather than explaining and talking’. These inscriptions replace some of the formal verbal communication between the scientists. In addition, new writable boards have been added to the lab, as the existing ones are all in use, for different purposes. As scientists cannot write directly on the white walls, Lewis and his colleagues have bought white rolls and stuck the hand-made white boards on the wall using red sticky tape, creating another surface on which to write and scribble formulae and visual instructions. The work spent to create more writable surfaces and to add inscriptions on the existing ones ultimately speeds up the course of lab work.

Further down is a plan of what ‘each section of the lab does’ drawn skilfully and placed in a user-friendly way on the wall behind the sink; the diagram shows the different areas (i.e. preparation, testing, analyses, etc.) and helps lab users to figure out the lab organization; ‘it improves the flow of work’. Here, again, speeding up the lab work is accomplished through specific devices that might temporary slow it down, such as the sketched lab plan. I watch Lewis: he makes his sample, he prepares the foil, he coats the sample and dries it, he assembles and he tests. The other researchers’ samples are waiting to be coated. It is a circular routine that follows the diagram on the wall. STS research has shown how specific instances of writing occur as part of a sequence of actions in a laboratory project, and ‘how written reports, photographic displays, notes, and recipes are employed by lab members as materials in their performance of lab work’ (Lynch, 1985, p. 152). The NGI example extends further the role of writing as important for collaborative lab work. While it signals to a creative reconfiguration of space, as discussed also by Gieryn (2002), this specific type of writing, not isolated in the format of a written document, but inscribed in the material surfaces as the observable flow of the work itself is oriented towards improving the speed and efficiency of experimentation. It acts as an organizer of lab duties and responsibilities, rather than a tacit stage. It allows the material architecture to become a mediator (Latour, 2005) of lab work. It is an active participant in the course of action, facilitating, translating and modifying the researchers’ epistemic activities. As such it creates specific relations between samples, scientists, equipment, industrial partners, computer data and fume hoods and actively *spaces* all lab work. Both the writing and the additional inscription surfaces added show the acute awareness of researchers of the importance of increasing the flow of work to achieve quick results and applications. The material features of the lab replace some standard forms of speech: pedagogical instructions, technical inductions and on some occasions, official scientific discourse. They reconfigure the formal types of verbal utterances by making them economical, allowing more time for social discussions.

Some of the writing signals informal chats. On the top of another fume hood, high up on the white surface of it, are some handwritten Basque words and their translation into English. These are the traces of a postdoctoral researcher teaching her energy lab colleagues different Basque words, just for fun. Lewis, in return, teaches them some slang English words. As Lewis sums up his lab life: ‘[W]hat I do in the lab is run experiments, analyse data, discuss results and have a joke with the other people.’ That summary can also be read through the scripts and formulae drafted on glass, paper and wall surfaces throughout the lab. The rhythm is hectic, but the work atmosphere is convivial. As some ethnographies of day-to-day laboratory practices have demonstrated, ‘shop talk’ is inherent to science as a social activity and is an inseparable accompaniment to all phases of laboratory research (Lynch, 1985). In the energy lab the ‘incipient talk’ of scientists, both epistemic and social, is imprinted in the material settings; ordinary lab conversations leave marks in space, on walls and surfaces, which generate even more discussions. Thus, the interaction among scientists is far from silent and solitary; it is diversified by new verbal utterances mediated by the lab architecture: scientists comment on writings and add new scribbles that prompt different discussions. While some inscriptions speed up the course of research, others intensify the social bonds. The time saved to re-tell the obvious, the known, is invested in a more epistemically and socially efficient flow of lab work, speeding up its course in search of the unknown.

### Stop 3: The grey rooms

**Figure 3.**
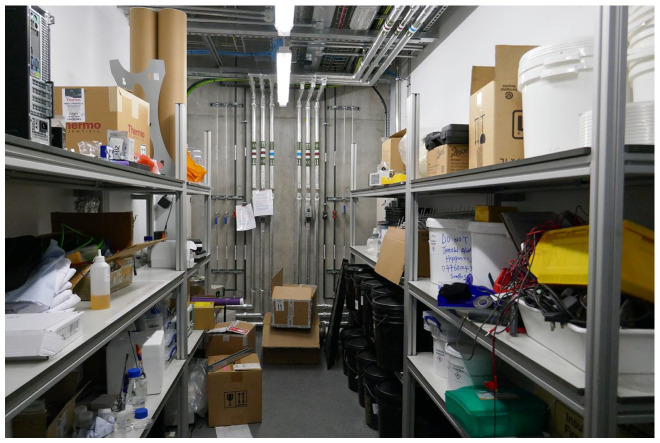
The grey room.

Lewis appreciates the fact that he does not need to carry a bottle of CO_2_ across the NGI building, as he does in the Chemistry Building where gas supply is not centralized. Everything is provided here, in every location, by a dedicated infrastructure that makes gases for a variety of different experiments always available. However, the hectic and flexible lab rhythm requires that more auxiliary equipment be installed. To respond to this need, expressed by scientists in the planning process of the building, special grey rooms were designed. They are two-meter-wide corridors adjacent to the labs (Figure 3). In these spaces, we can find air supply, electric and gas pipes, chilled water and isolators. The connection to gas lines and the power work is swiftly done through the grey rooms. Physical proximity allows the installation of new apparatuses to be done easily from there without the need for technicians to enter the lab and interrupt lab work.

Having grey spaces between the labs is something that a lot of the scientists have not experienced before. Most traditional science buildings designed in the 1960s can barely adapt to the single but changing disciplines they serve (Leslie, 2010). Their obsolescence emerges in my ethnography as participants repeatedly compare them with the NGI, as they go back and forth between buildings on the campus of Manchester University: the Schuster Building housing physics (built in 1967), the Material Sciences Tower (1968) and the Faraday Building now housing biochemistry (1967). If a research team in one of these buildings needs a hydrogen/argon mixture, a cylinder with such a mixture is provided and gets installed in their lab. Yet, as soon as a cylinder is introduced in the lab, on technician Christopher’s depiction of the setting, ‘it does become quite messy quite quickly, wires everywhere, etc’. At the NGI, in contrast, the cylinders disappear from the lab landscape, thanks to the grey space. Instead of them, neat terminals for the house gases are available, all ready to go and ‘feed’ the labs.

In addition to the fact that they allow quick and flexible retrofitting of the lab, the grey rooms are important in terms of safety. As senior scientist Mark explains, ‘we can store chemicals and gases in a space which is not occupied by people’. Separating substances from people is important for health and safety reasons, as researchers in the lab do not have to share a room with gas canisters and storage cabinets. This allows them also to ‘keep all the noisy, dirty equipment away from the lab itself’ explains Stuart, and ‘thus, compressors, vacuum pumps, and other equipment are kept outside. They can poke them into the labs when needed.’ Storing chemicals and loud equipment out of the lab, but in close proximity, helps preserve the clean and pleasant working environment that we witnessed in the energy lab (stop 2). The grey area is also ‘a space for storing something which you don’t use regularly, and you don’t need to access every day’. Yet, the boxes we spot in the grey room of the energy lab are not always welcome, as they can create a fire hazard, warns operations manager Polly. This space does encourage scientists to ‘keep stuff’ and hold onto things: ‘[T]hey will fill all available space, and whatever the space.’ Thus, the material architecture performs a spacing through careful separation of flows of people, equipment and chemicals needed to sustain the swift course of graphene research in the lab, from those that are not immediately needed and are temporally disconnected, invisible, waiting to be activated in new relational dynamics.

The separations are also important for the technicians. Another technician, James, explains that all of the service equipment is placed in the grey rooms because ‘if we leave sometimes a chiller out with them [the scientists], and it has a controller, the manufacturer might not want us to adjust that. But that chiller, if it’s left out in the lab, some of the researchers may feel like they have the authority to adjust that, whereas it’s safer for us to leave it out of their sight, and then it doesn’t get adjusted.’ The grey space, thus, performs another important function in regulating the relationships between the manufacturer of lab equipment, technicians, scientists and the equipment itself; it distributes the control over the manipulation of lab apparatuses differently. In the lab, scientists feel that they are in their territory, and so are entitled to control the equipment; in the grey room, territory and power shifts to the technicians. The two rooms, although adjacent, connected and interdependent, reshuffle the responsibilities of technical and scientific staff and redefine the quality of connections to samples and machines. The grey space, thus, has this double function. On the one hand, it keeps some of the noise, dirt, messiness and chemicals far from the lab, to be able to protect the scientists, their experiments and the speed of work. On the other hand, it keeps the scientists away from special equipment so as to preserve the technology and the integrity of technicians and manufacturers with the same purpose – to ensure a sustained course of work. Thus, the architecture of the grey room performs an important *spacing* of scientific, technical and maintenance activities.

Let us follow Lewis as he visits the grey room of the energy lab. It is shared with the lab next door. ‘This is our side and that’s their side’ explains Lewis, pointing to the different shelves, and he goes, ‘we mainly use it for storage of equipment that we don’t use anymore, whereas the other group, they use it for storage of samples. As they are more of a chemistry lab and they make lots of samples, which they store in.’ The chemistry lab has been set up for research on the chemical modifications of 2D materials and their applications in energy storage. Occasionally, Richard, who manages the energy lab and organizes the purchase of equipment, can put some samples in there as well, but his team never creates nearly as many as the neighbours in the chemistry lab. Sharing the grey space makes scientists curious about the on-going work of the next-door lab, and can sometimes result in collaborations. The energy team usually uses the samples in two days: ‘Things are never left aside for weeks’, clarifies Lewis, because of the way their experiments are run. If a sample is made and left for two weeks, it would not be good anymore. That is why everything is done quickly. If they start an experiment on Monday, they have the final device by Wednesday or Thursday and then they test it on Friday or over the weekend. This explains why the energy team samples are only stored for short periods in the grey room in contrast with the chemistry ones. Not a simple storage space, but a spacing device, the grey room is where the contrasting rhythms of work of the two labs can be observed. Its spacing potential depends on the quality of connection between apparatuses, samples, gases, scientists and technicians needed for energy or chemistry work. While scientists share labs, labs share grey rooms for samples and equipment. The collective effort permeates all aspects of work. Moreover, allowing samples and equipment from different labs to co-exist, the grey space can also incubate further fusions between chemistry and energy and foster new graphene applications.

To ensure that both the lab and the grey space function smoothly, Lewis needs the technician Christopher, who takes care of the house vacuum system, the process cooling system and the ultra-pure water; he ensures that all of the gases, the air, water and electrical supplies needed for experiments can be ‘injected’ straight into the labs. He keeps some cylinders connected in the grey space, fitted into a cabinet and securely locked. When special equipment is needed in the energy lab, Christopher takes the gas, water or electricity connections needed from the terminals through the wall and connects the cylinders in the grey rooms through 200-millimetre ports at the bottom of the walls; through these ports he can feed the pipe work without drilling through the walls. The technician’s work only affects a particular type of equipment, keeping minimal downtime, without creating disturbances so that researchers can carry on smoothly with their work. Accommodating all technical work of supply and servicing, the grey spaces illiminate the obstacles to sustaining the speed of graphene research in the lab. Here again, the material architecture of the grey room saves precious lab time. Connecting and disconnecting equipment and gases contributes to the work of spacing, just as humans, walls and inscriptions do in the lab.

**Figure 4.**
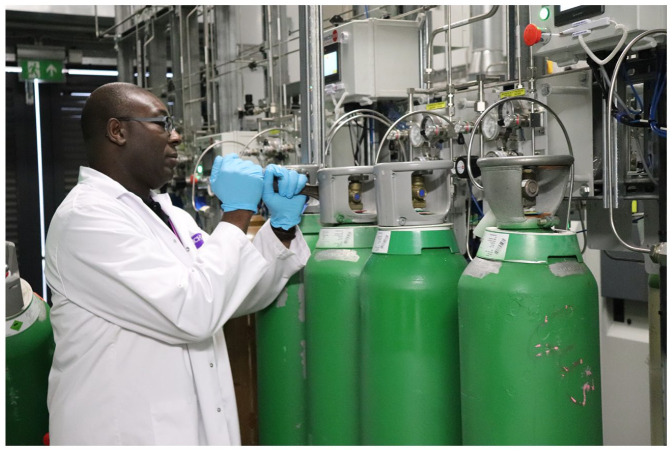
The gas room.

The work of another technician, Chris, who is in charge of the gas room (Figure 4), ensures the smooth and uninterrupted functioning of the entire infrastructure of ‘pipes leading through the ceilings and through the floors that go to various laboratories and clean rooms for the lab users to have access to gases’. Chris is monitoring the gases, and is also in charge of the ordering, storage, transportation and installation of cylinders. Together with other technicians, he ensures that there are no interruptions in the workflow. Commenting on their role, Chris says, ‘sometimes we are the unsung heroes because if we don’t do our work, they [the researchers] won’t be able to do any experiments’. Often Chris and Christopher are greeted in the morning with a problem that they are required to fix immediately. The flow of lab work is to be restored. As the utilities are constantly renewed, gases are filled and systems are updated, the course of graphene research runs unstoppably. Interruptions must be avoided, and the pace is maintained and cared for by the army of (commonly invisible) technicians. The flow of gases, interconnected alarm signals and safety features are continually watched through the Building Monitoring System to ensure the beat of graphene work never stops.

### Stop 4: The writeable walls

Taking a break from lab work, Lewis grabs a coffee. He stops in the atrium on the second floor and sits on a comfortable armchair facing the writable walls. Black PVC material covers the walls and encourages scientists to pause and write while chatting. Writing is another way, for them, to share insights, but writing also is a means of thinking through the exposure to other thought paths. The regime of visibility and exposure is reinforced here by the design of the writeable walls. This informal setting enables groups to mix with other groups. Scientists sit in a ‘café’ environment. Comfortable sofas contribute to the relaxed atmosphere. Rahul is convinced that ‘there might be aspects of work that are worth discussing while sitting in a sofa, in a more relaxed atmosphere’, as it might be helpful in trying to get the message across. This material arrangement affords a different epistemic and social exchange, compared to the communication in the offices or in the conference rooms.

**Figure 5.**
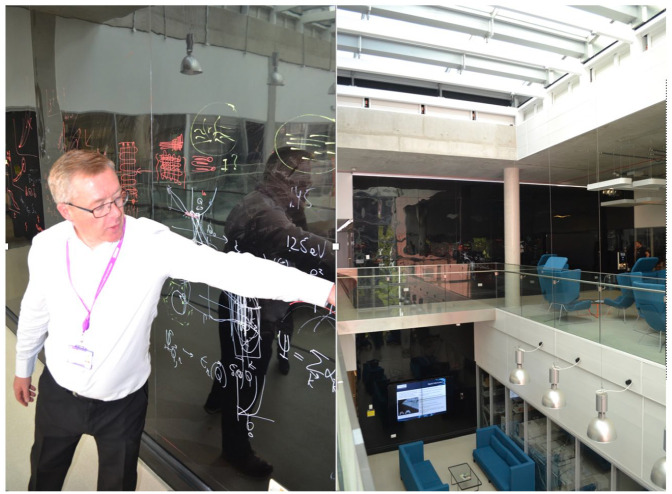
The writeable walls in the atrium.

A meeting of the theoretical physics group is in progress. Lewis stays for a little while. Their formulae are all over the wall (Figure 5) and they translate as: ‘[T]hese are two crystals aligned with each other. And what happens when you rotate them with respect to each other?’ There is also a structure of a moiré pattern being drafted. Researchers from other areas, experimental scientists who are less theoretically inclined, come along with coffees, sit down and listen. A few moments later in the same space, a company holds a presentation with industrial people working in the NGI; the meeting is open, and other interested people join as well. In this case, the writing shows what graphene and the industry is about; technical readiness level graphs showcase where everything sits in the life of a product. The walls are interactive and often change content: ‘[E]veryone’s thoughts are on the wall, and they get scrubbed off on a monthly basis.’ They follow the rhythm of current research themes and discussions. ‘The walls do something’, scientists repeat; they somehow intervene in the group meetings. Speeding up communication, reconfiguring the interactional orders of epistemic and social nature, connecting bodies and formulas in different relational configurations, the walls act as powerful spacing mediators. Facilitating epistemic exchange, and sometimes translating and modifying entire arguments, they actively participate in the course of graphene research. As the social areas lack fixed walls – except the writable ones – we witness in all these situations how the open architecture melts the rigid boundaries supposedly erected between theory and practice or science and industry; it facilitates collaborative learning and exchange across different levels. Temporarily slowing down to finish his coffee and take part in discussions around the writeable walls, Lewis will speed up again.

**Figure 6.**
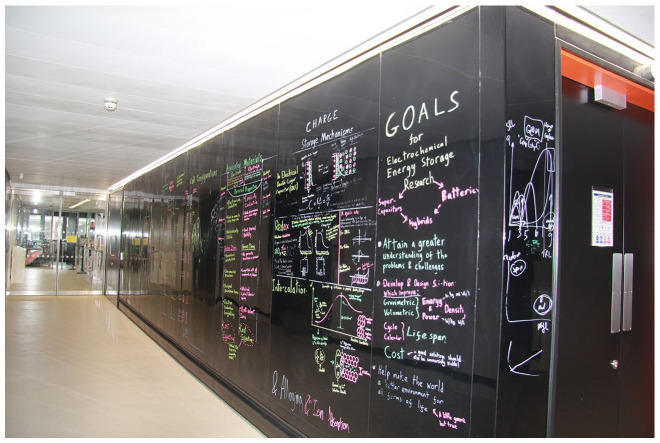
The writeable wall in the energy lab corridor.

Coming back to the corridor of the energy lab, he stops in front of the writeable walls there (Figure 6). Beautifully presented colourful diagrams and charts fill in these walls. The information is not sensitive in terms of intellectual property rights, as the work has already been published. These inscriptions are ‘a representation of what is going on in the lab’. They are used as a means of communicating current research projects to other groups, potential clients and collaborators. These walls are the business card of the lab. As the building is now a landmark in Manchester and guided tours are organized for the general public, the walls also protect the lab by preventing interested visitors from entering it and interrupting the work; everything they need to know is written on the walls. Lewis and his colleagues take responsibility for how they reflect the current lab work, for their maintenance and cleaning, whereas the walls in the social space are taken care of by everyone. Thus, walls operate differently: In the social space they serve the process of mutual sharing of recipes, rough thought paths and work in progress; they are active participants in the collective crafting of knowledge. Here, they are projective surfaces of crafted knowledge, a barrier impeding the access to the lab. In both cases, they support different working habits, afford and materially configure distinct conditions of visibility, mediate the work of making graphene research visible and perform the spacing of the social practices of all NGI dwellers.

The house attendants have specific instructions to not touch the walls, although they are often tempted to clean the writings. One, Gordon, explains: ‘[O]ur temptation is to clean the boards, but this might be somebody’s research!’ Another, Daniela, who worked in the Shuster Building eight year earlier, remembers accidentally cleaning a board that was not meant to be cleaned: ‘[T]here was a professor doing his research and all his notes were on the board and he wanted to keep them, look at them and then write again, look at the new notes and then write again, and keep them all. But I deleted them. It was horrible. So, I am scared of boards now. This professor was doing research for six months and all his work was on the board and vanished.’ But, here, both Daniela and Gordon know they cannot rub the walls around the NGI building, as they are meant to accommodate writings that contribute to graphene discussions. They speed up or slow down the machine of applied research. As with the house attendants, the building instils new habits in the scientists. In the traditional campus buildings, they cannot write on the corridor walls even when there are dedicated whiteboards; there, the expectation is that in the shared spaces one can pass messages but can rarely start a scientific discussion. In contrast, at the NGI the corridors can host discussions around quickly drafted formulae; the walls have eyes and minds and stimulate group thinking. Only occasionally do scientists happen to use the writable walls to leave a message, following old habits picked up in the other buildings – for example ‘gone to lunch and will be back at half one’. Sometimes, there may be other content that is not strictly related to the science happening inside the building – jokes, poems, announcements, etc. The walls become temporarily a projection of everything that goes in a scientific mind and as such they can also happen to slow down the speed of research. That is why writing that does not include ‘genuine thought paths’ or that does not relate to graphene is experienced as a form of ‘vandalism’, going against the building programme. Thus, just as transparency is not always experienced as efficient, for it eradicates all privacy, the exposed and accessible nature of the writable walls makes them vulnerable and leads to precarious practices. Both the danger of erasing valuable research content and that of adding irrelevant content is immanent to their daily use. They slow down the hectic course of graphene work, so it can accelerate again. Far from being a passive artistic decor, the walls perform an active spacing by bringing together actors with variable ontologies: scientists, inscriptions, cleaners, poets, detergents, jokes and formulae. As an extension of the collective minds of research and industry groups, they accelerate the epistemic exchange while also strengthening the social bonds.

While there is no downtime for the accelerated speed of graphene research, scientists need to slow down for a little longer than the quick moment needed to scribble a joke on the wall. Lewis’s breaks are often dictated by the nature of the lab work. If a break fills in the waiting time for an experiment in the energy lab, rather than interrupting the course of work, it can speed up the process as a whole. Sometimes, Lewis is just tired, and he can stop the process and go out on the terrace to enjoy lunch with the other researchers. Slowing down for a little longer, they contemplate the wild roof garden, breath fresh air, eat, chat, enjoy the nice view and the shy Manchester sun. Re-energized, they rush back to their labs or to the clean room, impatient to see the results from the morning experiment, speeding up again.

## Conclusion

Following the rhythm of scientific dwelling in its course in the hybrid lab of the National Graphene Institute in Manchester, I ask two questions that might indicate pathways for future inquiry on science architecture.

*What defines the hybrid lab?* The purpose of the hybrid lab goes far beyond the task of encouraging creative sociability arising out of unpredictable interactions and a ‘buzz’ of continuous conversation oriented to ‘transactional knowledge’. Interaction is not the ultimate goal, as witnessed here. Instead, scientists and industry people share spaces and equipment, rub shoulders at the lab benches, read results together or write on the black walls, and thus gradually change working habits. This also implies the cohabitation of diverse lab settings and technologies and their distinct hazards. It is at the level of these material practices that another type of hybridization and exchange transpires and crafts a type of relationship that is ontologically different from the subjective communication of ideas. Therefore, it is paramount to understand how all design facilities and support infrastructure in the hybrid lab contribute to the swapping of recipes and thought paths. The hybrid lab is not a state-of-the-art media company (Klonk, 2016), but a complex machine creating new ontological fusions that serve applied science.

Following the application-driven nature of graphene research, I identified three distinctive features of the hybrid lab. First, while we know that science is, at once, public and private (Gieryn, 1998), contemporary science shifts the balance between public and private places. Yet, if the work of modern science is an oscillation between intense communal interaction and solitude, at the NGI, the collective effort is present in all spaces, co-existence prevails, isolation becomes virtually impossible. Second, the hybrid lab also manages the juxtapositions of the visible and invisible in a distinctive way. If the modern laboratory renders natural objects visible, it makes the observing practices of scientists invisible to all but the few knowing experts. Similarly, the hybrid lab creates enhanced environments where it becomes possible to see things not visible elsewhere, but at the same time it renders visible the work of making visible, the thought processes: writing walls, façade formulas, transparent lab doors and partitions, inscriptions on the fume hood. Its design reinforces epistemically and socially the conditions of visibility and emphasizes the work of rendering its shifting boundaries conspicuous. The confidence in applied graphene research stems from this ubiquitous observability. Third, the hybrid lab highlights the work of spacing, and how spacing, timing and acting are to be combined with different intensities and speeds. Although speed and the construction of tempo (Gieryn, 2008; Stephens, 2012) are important features of the modern lab, in the hybrid lab it is the intensity of time and space that defines their deeper definition, depending on the quality of connection with other actors: walls, apparatuses, samples, technologies, partitions, people and infrastructure. Every design feature of the NGI building, as witnessed here, matters for graphene research, as it constantly accelerates the course and mediates the rhythm of experimentation. Even the ‘breakout’ spaces that are supposed to provide quality time produce a detachment that accelerates further the pace of research. A number of practices slow down research (producing additional inscriptions on lab surfaces, drinking a coffee, joining a discussion of the theory group, writing a joke on the wall, etc.); yet, they subsequently contribute to speeding it up. The labs are adapted quickly, the groups are formed and disbanded, the gas infrastructure never sleeps. The building operates as a machine that intensifies graphene work and actively enables scientists to explore a multiplicity of ways of being and generating scientific knowledge.

*How should we study the hybrid lab?* To capture the specificity of the hybrid lab, we need an approach to scientific architecture that grasps the multiple regimes of visibility and the various speeds of epistemic and social exchange. Bridging the divide between the technical performance and the human interface of science labs, it is vital to devise new types of longitudinal enquiries by paying equal attention to the complexity of the design facilities (the labs, the gas rooms, the grey spaces, the utilities blocks, the mechanical workshops and the ways of servicing them) and the variability of human experiences that they enable. All these facets of scientific dwelling contribute to seamless experimental work. It is paramount for STS scholars to attend to changes over time in how buildings and practices get interwoven. If the largest question for studies of the architecture of science has been to explore the relationships between the building and the shaping of scientific identities, we can see a new concern. A pragmatist one. Nothing is fixed in the hybrid lab and none of its spatial arrangements can reflect identities. It is instead important to unravel how its architecture translates, rearticulates and further contributes to the changing dynamics of applied science.

This study attempted to capture the work of spacing and how it affects graphene research. None of the NGI spaces, as witnessed here, are passive decor; they are, rather, active dispatchers of activities, modulators, incubators and spacing devices. The lab facilitates the organization of duties and the efficient workflow, the grey room enables separations between equipment and people, chemicals and humans, the social space encourages writing and thinking together. Following scientists, industry people, building managers, experimental officers and technicians, we see that the spaces and times of graphene research appear as consequences of the ways in which bodies, samples, equipment and partitions relate to one another. They express some specific relations between the entities themselves, and generate as many spaces and times as there are types of relations. Timings and tempos depend on ontological difference. The entities needed for scientific activities proliferate; the ways they make time and space vary. The speed of work is reliant on the obedience or the recalcitrance of all these entities. Thus, space is not a passive container that can be filled in with activities but is rather generated by the work of spacing. Walls, glass, writing surfaces, lab equipment, gas cylinders and samples all take part in this work and offer peculiar architectural ways of folding times with different tempos.

This raises a related question: *Who, and what, is to be included in the analysis of science architecture?* My study captured a number of voices from an array of participants commonly excluded from studies of scientific design. In addition to the usual actors encountered – the scientists and the architects – I followed lab technicians, facility managers, gas room and storage room technicians, house attendants and porters. Each of them works alongside the scientists to support their research, and assist, in an almost imperceptible way, the maintenance of the NGI’s high-spec infrastructure. Just as we should not allow the work of lab technicians to remain invisible (Mukerji, 1990; Shapin, 1989), we should also acknowledge the importance of all sorts of practical work contributing to the making of scientific knowledge. These are, as the storeroom technician Chris puts it, the ‘unsung heroes’ of lab work and their contribution to laboratory life needs further attention. A number of ‘unsung’ nonhuman actors join Chris as well: argon, cylinders, fume hoods, walls and surface inscriptions, alarms and building monitoring systems. Future studies of laboratories might benefit from considering their crucial mediating role.

The hybrid lab generates a new dynamic of innovation. As laboratories are integrally a part of their times and places, further pragmatist investigations of ‘labs as social and cultural infrastructures’ (Kohler, 2008, p. 764) and the spacing of research can shed light on the workings of contemporary applied science.
